# Age-Related Response Bias in the Decoding of Sad Facial Expressions

**DOI:** 10.3390/bs5040443

**Published:** 2015-10-27

**Authors:** Mara Fölster, Ursula Hess, Isabell Hühnel, Katja Werheid

**Affiliations:** Department of Psychology, Humboldt-Universität zu Berlin, Rudower Chaussee 18, Berlin D-12489, Germany; E-Mails: ursula.hess@psychologie.hu-berlin.de (U.H.); isabell.huehnel@hu-berlin.de (I.H.); katja.werheid@hu-berlin.de (K.W.)

**Keywords:** response bias, stereotypes, decoding accuracy, emotion attribution, facial expressions, aging

## Abstract

Recent studies have found that age is negatively associated with the accuracy of decoding emotional facial expressions; this effect of age was found for actors as well as for raters. Given that motivational differences and stereotypes may bias the attribution of emotion, the aim of the present study was to explore whether these age effects are due to response bias, that is, the unbalanced use of response categories. Thirty younger raters (19–30 years) and thirty older raters (65–81 years) viewed video clips of younger and older actors representing the same age ranges, and decoded their facial expressions. We computed both raw hit rates and bias-corrected hit rates to assess the influence of potential age-related response bias on decoding accuracy. Whereas raw hit rates indicated significant effects of both the actors’ and the raters’ ages on decoding accuracy for sadness, these age effects were no longer significant when response bias was corrected. Our results suggest that age effects on the accuracy of decoding facial expressions may be due, at least in part, to age-related response bias.

## 1. Introduction

Facial expressions play a central role in the quality of interpersonal interactions, conveying important information on the emotional states of our interaction partners [1]. Erroneous interpretations of facial expressions may lead to misunderstandings and impair the quality of interactions. For example, if a sad expression is misinterpreted as angry, conflict may result. Recent evidence suggests that the decoding of facial expressions is influenced by the ages of both the observer (“*rater*”) and the sender (“*actor*”). Specifically, it appears to be more difficult to decode the emotional facial expressions of older actors than those of younger actors (e.g., [2,3]); it also appears to be more difficult for older raters to decode facial expressions than it is for younger raters (see [4] for a review).

Furthermore, age may influence which emotions are attributed to facial expressions (*cf.* [5]). With respect to the age of actors, social stereotypes of aging may lead to more frequent attributions of emotions corresponding to these stereotypes and to fewer attributions of emotions contradicting these stereotypes [6]. With respect to the age of raters, age-related differences in experienced emotions may lead to different patterns of attribution for older *versus* younger raters [7]. Such response bias, that is the unbalanced use of response categories, may at least partially explain the aforementioned age effects regarding the degree to which facial expressions are accurately decoded (*decoding accuracy*) [8]. Specifically, decoding accuracy may be overestimated for those emotions that are frequently attributed and underestimated for those emotions that are infrequently attributed. Nevertheless, the bulk of the previous research has not taken such bias into account, and has used raw hit rates, that is, simple percentages of correct answers [9,10], or error rates [11] as outcome measures. Wagner [12] has argued that the use of such data may lead to an increase in Type 1 errors, particularly for spontaneous expressions (which result in lower accuracy rates) because any category that is used disproportionately often will appear to be more accurately decoded. In the extreme case, where, for example, a rater labels all expressions by older actors as sad, sadness would be decoded with 100% accuracy. The aim of the present study was to examine the influence of age-related response bias on the accuracy of decoding facial expressions.

### 1.1. Age-of-Actor Effects on Facial Expression Decoding

Older actors’ facial expressions tend to be decoded less accurately compared with younger actors’ expressions [2,3,9,10,13,14]. Several mechanisms may explain this result: age differences in expressivity [6], a preference for younger faces [9], lower expertise among raters for older faces [15], differences in the visual scanning [10,16] or the neural processing of older *versus* younger faces [17,18], and age-related structural changes in the face that reduce the signal clarity of expressions [2]. In line with this last explanation, the emotions that actors intended to show were rated as less intense, but the emotions that actors did not intend to show were rated as more intense on older faces [2,3].

Additionally, since individuals use stereotypes about social groups when decoding ambiguous facial expressions displayed by strangers [19], social stereotypes of the elderly may influence the attribution of emotions. With respect to the content of these stereotypes, older adults were evaluated more negatively in terms of integrity, defined as expectations for the future (e.g., optimism *versus* pessimism, hopefulness *versus* dejection, and happiness *versus* sadness) [20]. One might expect this stereotype to increase the frequency of attributing sadness and reduce the frequency of attributing happiness to older adults. Furthermore, wrinkles and folds and the sag of facial musculature may be misinterpreted as emotional expression [2]; for example, down-turned corners of the mouth may be mistaken for sadness.

Indeed, it appears that sadness is the emotion most likely to be associated with older faces, whereas happiness is more likely to be associated with younger faces. Thus, Bzdok, *et al.* [21] found a negative association between perceived age and perceived happiness of faces. Likewise, faces displaying a happy expression were perceived as being younger than faces displaying a fearful, angry, disgusted, or sad expression [22]. Furthermore, older faces were more frequently described as expressing sadness [6].

If such attribution bias is not statistically controlled, it may distort age-of-actor effects on decoding accuracy. For example, decoding accuracy for older actors may be overestimated for sadness and underestimated for happiness. Nevertheless, although some studies have examined response bias related to the age of the rater [23,24], no previous studies have, to our knowledge, examined response bias related to the age of the actor or assessed its role with regard to decoding accuracy using data that were corrected for response bias. Therefore, our aim was to investigate whether sadness is more frequently attributed to older actors and happiness to younger actors, and whether such response bias influences age-of-actor effects on decoding accuracy.

### 1.2. Age-of-Rater Effects on Facial Expression Decoding

Older raters have lower raw hit rates than younger raters (see [4,25] for reviews); however, this decline may be restricted to negative facial expressions [9]. These effects have been explained in terms of the socioemotional selectivity theory (SST) [26], which proposes that older persons are, due to their limited future time perspective, inclined to engage in tasks related to their own emotional balance and well-being. Younger persons, in contrast, favor information seeking over emotionally rewarding goals and thus may be more inclined to attend to the negative emotional states of others [27]. These motivational differences may also bias the attribution of emotions due to age differences in selective attention to cues that signal positive or negative experience [3]. In line with this assumption, older raters attributed fewer negative emotions to facial expressions [3,7,8], and the age-related decline in decoding accuracy for sadness was statistically explained by an age-related decrease in negative affect [28]. Thus, Bucks, Garner, Tarrant, Bradley and Mogg [8] argued that age differences in decoding accuracy may reflect differences in response bias rather than differences in perceptual discrimination.

However, in some studies, age-related response bias was not observed, and age differences in decoding accuracy remained significant when response bias was controlled [23,24]. One alternative explanation for the relatively preserved ability to decode happiness is that happiness may be easier to decode than negative emotions are, particularly when it is the only positive response option [25]. Happy facial expressions may be easier to decode because they involve distinct facial movements (smiling) whereas negative emotional expressions share some movements [29]; for example, sadness, fear and anger expressions involve lowering of the brows [30]. As age differences in cognitive tasks increase with task difficulty [31], age differences in decoding accuracy for negative emotions may reflect general age differences in information processing capabilities. Hence, the role of age-related response bias remains unclear and deserves further investigation.

### 1.3. Own-Age Effects on Facial Expression Decoding

The majority of previous studies did not find an own-age advantage, but rather higher accuracy (*i.e.*, higher raw hit rates or lower error rates) for younger as opposed to older actors in both rater age groups ([9,10,13], but see [11] for an exception). Furthermore, although older adults may have more favorable stereotypes of aging (see [32] for a review), the content of age-related stereotypes is largely comparable between younger and older raters [33], suggesting similar influences of age-related stereotypes on emotion attributions. Thus, we did not have any specific expectations concerning own-age effects on response biases and their influence on decoding accuracy.

### 1.4. The Present Study

Most previous research investigating age effects on decoding accuracy used static images of posed expressions (see [11,14] for exceptions). In contrast, the present study used spontaneous dynamic facial expressions because some emotional information is encoded dynamically [34,35], and the use of spontaneous dynamic presentation circumvents the ceiling effects often reported for posed, static expressions, especially for happy faces [25].

The aim of our study was to extend previous research by examining whether age-related response bias distorts age effects on decoding accuracy. Age-related response bias was analyzed by comparing age effects for raw hit rates with age effects for unbiased hit rates corrected for the number of uses of a response category for each stimulus type, following Wagner [12]. Consistent with the stereotype of older adults as being lower in integrity, we expected sadness to be attributed more frequently to older actors and happiness to younger actors. According to the SST, we expected older raters to attribute more positive and fewer negative emotions than would younger raters. We expected both age-of-actor and age-of-rater effects on decoding accuracy to be reduced when response bias was controlled.

## 2. Method

### 2.1. Participants

Thirty older raters (*M* = 70.33 years, *SD* = 3.60, 15 male, 15 female) and thirty younger raters (*M* = 23.93 years, *SD* = 2.99, 15 male, 15 female) participated in the decoding study. The participants were screened for psychiatric and neurological disorders and were recruited via an online participant database at the Humboldt-Universität zu Berlin, by means of flyers distributed in a retirement home, pharmacies, a library, and senior recreational facilities, as well as via an e-mail-newsletter of the Third Age University of Humboldt-Universität zu Berlin. Raters received either course credit or remuneration of 10€ per hour for participating. The study was approved by the ethics committee of the Department of Psychology, Humboldt-Universität zu Berlin, Reg.-No 2010-06.

The two rater-age groups did not differ in crystallized intelligence, *t*(56) = 1.29, *p* = 0.203, as assessed by the Wortschatztest (WST) [36], a German vocabulary test in which a target word has to be identified from among five pseudo-words^1^. However, younger raters had more years of education (younger raters: *M* = 12.80, *SD* = 0.49; older raters: *M* = 11.22, *SD* = 1.86); *t*(25^2^) = 3.96, *p* = 0.001, and, as expected, showed higher levels of fluid intelligence (younger raters: *M* = 30.20, *SD* = 3.84; older raters: *M* = 19.28, *SD* = 4.64), *t*(57) = 9.88, *p* < 0.001, as assessed by the reasoning subtest (subtest 3) of the Leistungsprüfsystem (LPS) [37], in which non-matching figures have to be identified from among logically related figures.

### 2.2. Current Affect

To test whether younger and older raters differed in current affect, according to predictions by the SST, we used the Positive and Negative Affect Schedule (PANAS [38]; German version [39]) with short-term instruction (“How do you feel right now?”). As expected, compared with younger raters, older raters reported higher positive affect (older raters: *M* = 32.38, *SD* = 6.52; younger raters: *M* = 27.83, *SD* = 6.56), *t*(57) = 2.67, *p* = 0.010, and lower negative affect (older raters: *M* = 11.28, *SD* = 1.56; younger raters: *M* = 13.95, *SD* = 3.99), *t*(38^2^) = 3.41, *p* = 0.002.

### 2.3. Age-Related Stereotypes

We used the Aging Semantic Differential (ASD [40]; German version [41]). Raters indicated how they generally perceived older adults and younger adults on a seven-point semantic differential scale. The literature supports four ASD factors: instrumentality (*i.e.*, adaptability, activity), integrity (*i.e.*, personal satisfaction, optimism, peacefulness with oneself), autonomy (*i.e.*, autonomy, self-sufficiency), and acceptability (*i.e.*, sociability) [20]. Mean scores for these four ASD factors were computed, with higher scores on each factor representing more negative evaluations. Analysis by means of 2 (age-of-rater) × 2 (age-of-target) repeated-measures ANOVA (analysis of variance) on these scores confirmed that, as predicted, the older target group elicited more negative evaluations than did the younger target group with respect to integrity (younger target group: *M* = 3.15, *SD* = 0.82; older target group: *M* = 4.07, *SD* = 1.00), *F* (1, 57) = 35.60, *p* < 0.001, η_p_^2^ = 0.384. Furthermore, in line with previous research [20], the older target group elicited more negative evaluations with respect to instrumentality (younger target group: *M* = 2.88, *SD* = 0.80; older target group: *M* = 4.29, *SD* = 0.84), *F*(1, 57) = 91.05, *p* < 0.001, η_p_^2^ = 0.615, but more positive evaluations with respect to autonomy (younger target group: *M* = 3.92, *SD* = 0.87; older target group: *M* = 3.21, *SD* = 0.91), *F*(1, 57) = 24.74, *p* < 0.001, η_p_^2^ = 0.303.

### 2.4. Stimulus Materials

Short videos depicting younger and older actors talking about past real-life events were created using the “social sharing paradigm” established by Christophe and Rimé [42]^3^. Previous research using this paradigm has shown that people experience a congruent emotional state when sharing emotional events in a social setting [43]. Thirty older (*M* = 72.37 years, *SD* = 6.48; 15 men, 15 women) and thirty younger (*M* = 24.47, *SD* = 3.17; 15 men, 15 women) adults were recruited as actors using the same recruiting procedures described above. For each of five target emotions (fear, disgust, happiness, sadness, anger), actors were asked to select two events in which they had experienced the target emotion from a list of prototypical everyday emotion-eliciting events. The actors in the present study were asked to write short summaries of the chosen events to refresh their memories and then to describe the events (as well as one neutral event) in front of the camera. After each shooting, actors filled out an emotion intensity questionnaire (except in the case of the neutral situation), describing how they had felt when thinking about the event they had just described. The answers in the questionnaire consisted of 15 items, three for each target emotion. The response scale ranged from 1 (not at all) to 7 (very intense).

**Figure 1:**
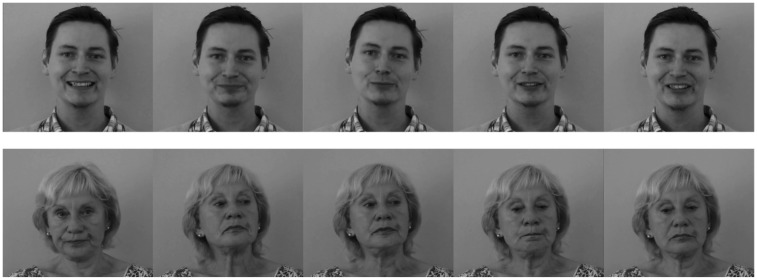
Sample frames of two video clips, showing one younger male actor expressing happiness and one older female actor expressing sadness.

The recordings were screened by two research assistants. Actors with distracting striking facial features (e.g., piercings, extremely heavy eyebrows), and those showing no discernible facial expression or systematically not looking into the camera were excluded. From the remaining recordings of 18 younger and 16 older actors, 20-s sequences were extracted containing the culminating point of the emotional episodes. The chosen sequences were converted to greyscale and cut into standardized image sections such that the lower edge of the image was aligned with the actor’s clavicle and the head appeared in the middle of the section. The video clips were presented without sound. Figure 1 shows samples of frames from two video clips.

For each narration, the mean intensity of experienced fear, disgust, happiness, anger, or sadness was calculated and analyzed by means of 5 (emotion scales) × 2 (age-of-actor) repeated-measures ANOVA for each target emotion. Significant main effects of emotion emerged for all target emotions (fear: *F*(3, 96) = 25.19, *p* < 0.001, η_p_^2^ = 0.441; disgust: *F*(3, 90) = 60.51, *p* < 0.001, η_p_^2^ = 0.654; happiness: *F*(1, 34) = 582.51, *p* < 0.001, η_p_^2^ = 0.951; sadness: *F*(3, 89) = 27.52, *p* < 0.001, η_p_^2^ = 0.478; anger: *F*(4, 120) = 50.66, *p* < 0.001, η_p_^2^ = 0.628). Post hoc tests revealed that the target emotions were always experienced significantly more intensely than were all non-target emotions (all *p*’s < 0.001).

A main effect of age-of-actor on the intensity of experienced emotions was only found for disgust, *F*(1, 32) = 7.56, *p* = 0.010, η_p_^2^ = 0.191, qualified by a significant emotion × age-of-actor interaction, *F*(3, 90) = 3.33, *p* = 0.026, η_p_^2^ = 0.094. Post hoc t-tests revealed that older actors experienced significantly more disgust (*M* = 6.17, *SD* = 1.07) than did younger actors (*M* = 5.15, *SD* = 1.65), *t*(32) = 2.11, *p* = 0.043, but they did not differ in the intensity of any other emotion. Furthermore, a significant emotion × age-of-actor interaction for anger, *F*(4, 120) = 2.47, *p* = 0.048, η_p_^2^ = 0.076, revealed that older actors experienced significantly more fear (*M* = 3.29, *SD* = 1.79) than younger actors did during anger situations (*M* = 2.00, *SD* = 1.18), *t*(30) = 2.43, *p* = 0.022. There were no significant differences for the remaining emotions.

### 2.5. Procedure

Raters were tested individually or in groups of two to three persons. After providing informed consent, raters completed the PANAS. The emotion decoding task was conducted by computer. After watching each video sequence, raters chose one emotion from a list of emotions in a forced-choice task. To enhance reliability, the emotions of contempt and surprise and the options “neutral” and “none of these” were provided in addition to the five target emotions, resulting in nine response options. To avert mental fatigue among the participants, three sets of video clips were created. The first two sets showed expressions by eleven actors, and the third by twelve actors. Actors were randomly assigned to the stimulus sets. Each rater viewed only one set. The sets were presented in three different randomized orders, with the restriction that there were never two adjacent sequences showing the same actor or the same emotion. After the decoding task, raters completed the intelligence tests and the ASD as described above.

### 2.6. Statistical Analyses

To examine the central question of the study regarding the influence of age-related response bias on the decoding of facial expressions, we followed the same three-step procedure that was used in previous studies to analyze the influence of response bias pertaining to the age of the rater on decoding accuracy [23,24]. First, we obtained uncorrected raw hit rates, *i.e.*, proportions of correct answers. A rating was considered correct when the rater chose the label corresponding to the target emotion. Age effects on raw hit rates were analyzed by a 2 (age-of-rater) × 2 (age-of-actor) × 5 (target emotion) repeated-measures ANOVA. Second, we obtained proportions of emotion attributions for each actor age group, *i.e.*, the frequency of attributions to each actor age group divided by the total number of rated videos for that group, and analyzed age effects on emotion attributions by a 2 (age-of-actor) × 2 (age-of-rater) × 9 (target emotion) repeated-measures ANOVA. Third, we calculated Wagner’s [12] unbiased hit rates by dividing the squared number of accurate answers by the product of the number of videos, and the number of responses in which that emotion was attributed to that actor age group. For example, the unbiased hit rate for disgust for younger actors was calculated according to the following formula:

(1) $$\text{HU}=\frac{{\left(\text{number}\;\text{of}\;\text{accurate}\;\text{disgust}\;\text{answers}\;\text{for}\;\text{younger}\;\text{actors}\right)}^{2}}{\left(\text{number}\;\text{of}\;\text{disgust}\;\text{videos}\;\text{of}\;\text{younger}\;\text{actors}\right)\times \left(\text{number}\;\text{of}\;\text{disgust}\;\text{attributions}\;\text{for}\;\text{younger}\;\text{actors}\right)}$$

Following the procedure suggested by Wagner (1993), unbiased hit rates were arcsine transformed. We conducted a 2 (age-of-rater) × 2 (age-of-actor) × 5 (target emotion) repeated measures ANOVA on these unbiased hit rates and compared them with the raw hit rates. Additional correlational analyses were conducted to analyze the relationship of age-related response bias with the integrity subscale of the ASD and current mood, as measured by the PANAS.

Interactions were followed up by direct comparisons. If the criterion of sphericity was violated, Greenhouse-Geisser corrections were applied and dfs were rounded to the nearest integer. For all statistical analyses, the alpha level was set at 0.05 (two-tailed). Data from one older female rater were excluded because of hit rates below chance level.

## 3. Results

### 3.1. Age Effects on Raw Hit Rates (Step 1)

The results of the age-of-actor × age-of-rater × target emotion ANOVA are displayed in Table 1. The facial expressions of younger and older actors were, on average, equally well decoded. A significant target emotion × age-of-actor interaction indicated emotion-specific age-of-actor effects. Results of follow-up analyses are displayed in Table 2. Sadness and fear were more accurately decoded for older actors, whereas disgust was more accurately decoded for younger actors.

**Table 1:** Results of ANOVAs on raw hit rates (left part) and unbiased hit rates (right part) as a function of age-of-actor, age-of-rater, and target emotion.

	Raw Hit Rate				Unbiased Hit Rate			
Source	*df*	*F*	*p*	η_p_²	*df*	*F*	*p*	η_p_²
Age-of-actor (AA)	1, 57	0.09	0.767	0.001	1, 57	0.02	0.901	0.000
Age-of-rater (AR)	1, 57	14.17	<0.001	0.199	1, 57	15.98	<0.001	0.219
Targetemotion (TE)	3, 199	85.76	<0.001	0.601	3, 168	78.33	<0.001	0.579
AR × AA	1, 57	5.56	0.022	0.089	1, 57	2.06	0.156	0.035
AR × TE	4, 217	3.05	0.020	0.051	3, 168	3.88	0.011	0.064
AA × TE	4, 228	19.69	<0.001	0.257	3, 173	9.67	<0.001	0.145
AR × AA × TE	4, 228	2.43	0.049	0.041	3, 173	2.05	0.107	0.035

Turning to the rater effects, a main effect of age-of-rater on raw hit rates emerged, mediated by a significant target emotion × age-of-rater interaction (Table 1). Follow-up analyses indicated higher hit rates for younger raters when decoding sadness, disgust, and happiness (Table 2).

With regard to age congruence, the significant interaction between age-of-actor and age-of-rater on raw hit rates was qualified by a significant three-way interaction among age-of-actor, age-of-rater, and target emotion (see Table 1). Follow-up analyses revealed significant age-of-actor × age-of-rater interactions for sadness, *F*(1, 57) = 4.84, *p* = 0.032, η_p_^2^ = 0.078, and disgust, *F*(1, 57) = 8.57, *p* = 0.005, η_p_^2^ = 0.131, but not for fear, *F*(1, 57) = 0.25, *p* = 0.619, η_p_^2^ = 0.004, happiness, *F*(1, 57) = 0.57, *p* = 0.452, η_p_^2^ = 0.010, or anger, *F*(1, 57) = 2.92, *p* = 0.093, η_p_^2^ = 0.049. Post hoc t-tests revealed that older raters achieved higher raw hit rates when decoding older (*M* = 0.35, *SD* = 0.17) than younger actors’ expressions of sadness (*M* = 0.14, *SD* = 0.15), *t*(28) = 5.30, *p* < 0.001, whereas younger raters’ accuracy for sadness did not differ between younger (*M* = 0.29, *SD* = 0.23) and older actors (*M* = 0.37, *SD* = 0.17), *t*(29) = 1.76, *p* = 0.089. When decoding facial expressions of disgust, both younger, *t*(29) = 6.47, *p* < 0.001, and older raters, *t*(28) = 2.61, *p* = 0.014, achieved higher raw hit rates when the actors were younger (younger raters: *M* = 0.39, *SD* = 0.20; older raters: *M* = 0.19, *SD* = 0.20) than when actors were older (younger raters: *M* = 0.14, *SD* = 0.19; older raters: *M* = 0.10, *SD* = 0.13), but this effect was more pronounced for younger raters than for older raters. Thus, there was a relative own-age accuracy advantage with respect to sadness and disgust.

**Table 2:** Means and results of simple effects analyses on raw hit rates (upper part) and unbiased hit rates (lower part) for each emotion as a function of (A) age-of-actor and (B) age-of-rater.

**(A) Age-of-Actor**							
**Emotion and Outcome Measure**	**Young Actors**		**Older Actors**				
	***M***	***SD***	***M***	***SD***	***F*(1, 57)**	***p***	**η_p_^2^**
Raw hit rate							
Fear	0.07	0.12	0.15	0.17	9.99	0.003	0.149
Disgust	0.30	0.22	0.12	0.16	42.23	<0.001	0.426
Happiness	0.62	0.26	0.59	0.24	1.23	0.273	0.021
Sadness	0.21	0.21	0.36	0.17	23.25	<0.001	0.290
Anger	0.26	0.18	0.28	0.18	0.40	0.530	0.007
Arcsine unbiased hit rate							
Fear	0.03	0.06	0.07	0.12	5.76	0.020	0.091
Disgust	0.26	0.23	0.11	0.16	22.55	<0.001	0.283
Happiness	0.39	0.24	0.45	0.29	1.81	0.184	0.031
Sadness	0.15	0.18	0.19	0.11	2.90	0.094	0.048
Anger	0.13	0.13	0.13	0.11	0.01	0.945	0.000
**(B) Age-of-Rater**							
**Emotion and Outcome Measure**	**Young Raters**		**Older Raters**				
	***M***	***SD***	***M***	***SD***	***F*(1, 57)**	***p***	**η_p_^2^**
Raw hit rate							
Fear	0.12	0.1	0.10	0.12	0.58	0.451	0.010
Disgust	0.28	0.17	0.15	0.14	9.39	0.003	0.141
Happiness	0.67	0.22	0.54	0.18	6.56	0.013	0.103
Sadness	0.33	0.16	0.23	0.12	5.63	0.021	0.090
Anger	0.26	0.15	0.29	0.15	0.70	0.408	0.012
Arcsine unbiased hit rate							
Fear	0.06	0.05	0.04	0.06	0.77	0.385	0.013
Disgust	0.23	0.16	0.13	0.13	5.96	0.018	0.095
Happiness	0.46	0.21	0.32	0.13	11.68	0.001	0.170
Sadness	0.18	0.11	0.14	0.10	1.75	0.191	0.030
Anger	0.13	0.08	0.10	0.07	1.76	0.190	0.030

### 3.2. Age-Related Response Bias (Step 2)

Based on previous research, we expected sadness to be more frequently attributed to older actors and happiness to be more frequently attributed to younger actors. Furthermore, we expected older raters to attribute negative emotions with lower frequency than younger raters would, but to attribute positive emotions with greater frequency.

**Figure 2:**
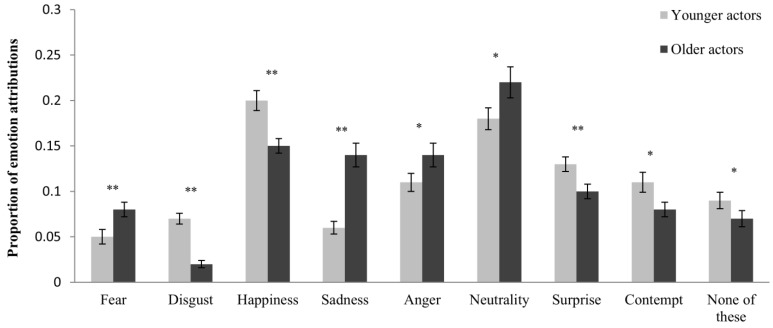
Proportion of emotion attributions separately for younger and older actors and each target emotion. Error bars represent standard errors of the mean. * *p* < 0.05, ** *p* < 0.01.

In line with our expectations, analysis of the response bias related to the age of the actor revealed a significant emotion × age-of-actor interaction, *F*(5, 283) = 13.88, *p* < 0.001, η_p_^2^ = 196. Results of follow-up analyses for each emotion are displayed in Table 3. As expected, sadness was more frequently attributed to older than to younger actors, whereas happiness was more frequently attributed to younger actors (see Figure 2 for means and standard errors). Disgust, surprise, contempt, and “none of these” were also more frequently attributed to younger actors, whereas fear, neutrality, and anger were more frequently attributed to older actors.

**Table 3:** Results of ANOVAs on proportions of emotion attributions as a function of age-of-actor (left part) and age-of-rater (right part).

	Age-of-Actor			Age-of-Rater		
Emotion	*F*(1, 57)	*p*	η_p_^2^	*F*(1, 57)	*p*	η_p_^2^
Fear	10.92	0.002	0.161	0.38	0.538	0.007
Disgust	64.84	<0.001	0.532	10.19	0.002	0.152
Happiness	22.24	<0.001	0.281	0.81	0.371	0.014
Sadness	28.99	<0.001	0.337	5.24	0.026	0.084
Neutrality	5.02	0.029	0.081	0.75	0.390	0.013
Anger	6.18	0.016	0.098	7.00	0.011	0.109
Surprise	11.73	0.001	0.171	5.29	0.025	0.085
Contempt	5.69	0.020	0.091	1.80	0.185	0.031
None of these	5.75	0.020	0.092	0.25	0.622	0.004

**Figure 3:**
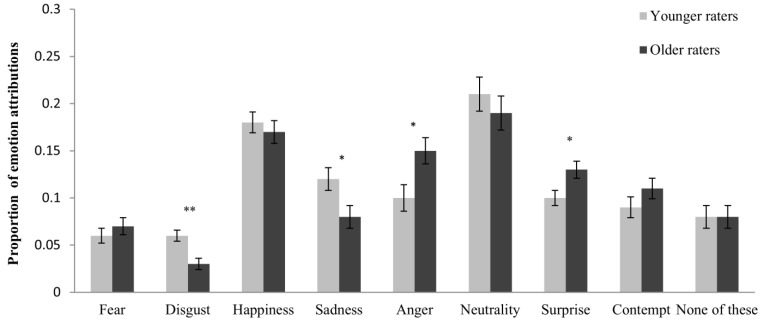
Proportion of emotion attributions shown separately for younger and older raters and each target emotion. Error bars represent standard errors of the mean. * *p* < 0.05, ** *p* < 0.01.

As expected, an age-of-rater response bias also emerged, as indicated by a significant emotion × age-of-rater interaction, *F*(5, 294) = 2.85, *p* = 0.015, η_p_^2^ = 048. Confirming the predictions of the SST, disgust and sadness were more frequently attributed by younger raters (see Table 3 and Figure 3). In contrast to the predictions of the SST, however, anger was more frequently attributed by older raters, as was surprise. The own-age effect for proportions of emotion attributions approached significance, *F*(5, 283) = 2.06, *p* = 0.071, η_p_^2^ = 0.035.

### 3.3. Unbiased Hit Rates (Step 3)

Unbiased hit rates, which were corrected for the number of attributions of the target emotion for each actor age group according to Wagner [12], were analyzed in the final step and compared with raw hit rates. The raw hit rates revealed no significant main effect of age-of-actor, but did yield a significant target emotion × age-of-actor interaction, indicating emotion-specific age-of-actor effects on unbiased hit rates (see Table 1). Unbiased hit rates resulted in a similar pattern, except with regard to sadness (see Table 2). For sadness, raw hit rates were significantly higher for older than for younger actors, whereas this difference was only marginally significant for unbiased hit rates. Furthermore, the age-of-actor effect size was considerably lower for unbiased hit rates compared with raw hit rates for sadness (see Table 2). Thus, the effect observed for raw hit rates was at least in part due to the more frequent attribution of sadness to older actors. Results did not differ between unbiased and raw hit rates for the remaining emotions. To further explore whether this response bias for sadness was due to the stereotype of older persons as lower in integrity, we calculated the correlation between the proportion of sadness responses attributed to older actors with the ASD integrity scores for older target persons; this correlation was not significant, *r* = 0.12, *p* = 0.380.

Turning to the age-of-rater effects, a significant main effect of age-of-rater emerged, as did an interaction of target emotion × age-of-rater, resembling the results for raw hit rates (Table 1). Follow-up analyses (see Table 2) revealed no significant results for sadness, in contrast to the aforementioned higher raw hit rates for younger *versus* older raters. Additionally, the age-of-rater effect size was substantially lower for unbiased hit rates of sadness than it was for raw hit rates of sadness (see Table 2). Thus, in line with the predictions of the SST, younger raters’ higher raw hit rates for sadness were due to a more frequent attribution of sadness by younger raters. Nevertheless, contrary to the SST-based account, the proportion of responses attributing sadness was not correlated with negative, *r* = 0.16, *p* = 0.230, or positive current affect, *r* = 0.09, *p* = 0.493. The remaining emotions yielded no significant differences between unbiased and raw hit rates with respect to age-of-rater effects.

Own-age effects differed between unbiased and raw hit rates. In contrast to raw hit rates, there was no two-way age-of-actor × age-of-rater interaction, nor was there a three-way interaction between age-of-actor, age-of-rater, and target emotion (see Table 1). This suggests that the own-age effects on emotion attribution reported above, although only approaching significance, may have distorted own-age effects on raw hit rates.

## 4. Discussion

In summary, the data supported our hypothesis that age-related response bias distorts age effects on decoding accuracy with respect to sadness. Although raw hit rates suggested higher decoding accuracy for sadness for older than for younger actors, and by younger than by older raters, these age differences were no longer significant when response bias was controlled. Furthermore, raw hit rates suggested an own-age advantage for decoding sadness and disgust, but this own-age advantage similarly vanished when response bias was controlled. Correlation analysis revealed that the age-related response bias for sadness was not related to the stereotype of lower integrity in older adults, nor was it related to age differences in current affect.

### 4.1. Age-of-Actor Effects on Facial Expression Decoding

Raw hit rates suggested greater accuracy in the decoding of sad expressions for older relative to younger actors, but this difference was no longer significant when response bias was controlled. Thus, this difference was at least in part due to a tendency to attribute sadness more frequently to older actors, in line with previous research [3,6]. Notably, this response bias was not related to corresponding age-related differences in the intensity of emotions experienced by the actors.

Moreover, as we further predicted, happiness was more frequently attributed to younger actors than to older actors, in accordance with earlier findings that happy faces were perceived as younger [21,22] and with reports of stereotypes of older adults as being less satisfied [20].

Self-report measures confirmed that the participants in our study held the stereotype of older persons as lower in integrity than younger individuals, that is, as being more pessimistic, dejected, and sad. Surprisingly, however, the proportion of sadness attributed to older actors was not statistically related to this stereotype. One possible explanation for this apparent discrepancy is that there may be a disassociation between explicit stereotypes of the elderly, as reflected by the self-report measures, and implicit stereotypes, as reflected in decoding bias. In fact, typically only moderate [44,45] or non-significant [46] relationships between implicit and explicit stereotypes have been observed. Thus, individuals may express socially desirable attitudes to reinforce a positive self-image, but their emotional attributions may nevertheless be biased, reflecting implicit stereotypes. As an alternative explanation for the lack of correlation between ASD measures and the proportion of sadness attributions that involved older actors, the more frequent attribution of sadness to older actors may be due to the fact that facial features of older actors resemble facial expressions of sadness (e.g., both may include down-turned corners of the mouth).

Additional response bias related to the age of the actor was found for disgust, surprise, and contempt, all of which were more frequently attributed to younger actors, and for fear and anger, which were more frequently attributed to older actors. In contrast to these results, previous studies found either no significant age-of-actor response bias [11] or a less frequent attribution of anger to older actors [6]. Thus, the overall pattern of age-of-actor effects on emotion attributions is still inconclusive and needs further investigation. We speculate that the pattern of response biases observed here may be related to the perceived differences in dominance between the two age groups. Because older people are perceived as being low in competence but high in warmth [47], they may be expected to be less apt to display emotions that are associated with greater dominance (which is closely related to competence), such as disgust [48,49], but more likely to display emotions that are associated with lesser dominance, such as fear and sadness [48,49]. However, the finding of more frequent attribution of anger to older actors, an emotion that is associated with high dominance [49], contradicts the predictions suggested by this account. Thus, there may be additional stereotypes concerning older adults’ emotional experiences that are not related to dominance. For example, the common aging stereotype of shrew/curmudgeon [33] may explain the more frequent attribution of anger to older actors. Future research should assess age-related stereotypes for a broader range of emotions and examine the relationships between these stereotypes and response bias in facial expression decoding.

Although response biases accounted for some age-of-actor effects on decoding accuracy, other age-of-actor effects remained significant when response bias was controlled. Thus, age-related response biases are unlikely to be the sole mechanism underlying age-of-actor effects on decoding accuracy. As an alternative mechanism, visual scan patterns may differ for younger and older faces. Thus, participants looked longer at the eye region of older than younger neutral faces, and longer at the mouth region of younger than older faces [16]. As the eye region is more important for fear, but not for disgust [50], this may explain the higher decoding accuracy for disgust in younger and fear in older faces. However, so far, this explanation is only speculative. Future studies examining decoding accuracy for different actor age groups while recording raters’ visual scan patterns may elucidate this point.

### 4.2. Age-of-Rater Effects on Facial Expression Decoding

In line with SST predictions, age-of-rater effects differed depending on whether unbiased or raw hit rates were used as the outcome measure. Raw hit rates suggested an age-related decline in the ability to decode sadness, but this age difference vanished when response bias was controlled. This implies that at least for sadness, age differences reported in the literature (see [4] for a review) may be due not to an age-related decline in the ability to decode sadness, but rather to the less frequent attribution of sadness by older raters. Thus, this finding reflects the use of an outcome measure that was not corrected for age-related response bias.

Also in keeping with SST predictions and with previous reports of older raters’ attributing fewer negative emotions [3,7,8], disgust was attributed less frequently by older than by younger raters. Furthermore, older raters exhibited higher scores for positive current affect and lower scores for negative current affect compared with younger raters. Thus, the actual experience of negative emotions may prime younger adults to attribute sadness more readily. Alternatively, older adults may be less motivated to attend to negative information than to positive information [3].

However, contrary to SST predictions, the proportion of sadness attributions was not statistically related to positive or negative current affect. Additionally, older raters attributed anger more frequently and had lower decoding accuracy for happiness compared with younger raters. Furthermore, age-related differences in decoding accuracy were not fully explained by response bias, as younger raters achieved greater decoding accuracy for disgust even when response bias was controlled. Thus, age-related motivational differences cannot fully account for the age-of-rater effects on response bias and decoding accuracy. Other possible mechanisms such as age differences in visual scan patterns [51,52] may also play an important role in explaining age differences in decoding accuracy.

This latter conclusion is consistent with previous findings that age-of-rater effects on decoding accuracy remain significant when response biases are controlled [23,24]. As a possible explanation for our discrepant results, age-related response biases have a stronger influence on decoding accuracy when spontaneous facial expressions are used and guessing rates are high [12], such as in the present study. In line with this explanation, raw hit rates were considerably higher in previous studies (on average higher than *M* = 0.80 [23,24]) than in the present study (*M* = 0.31).

### 4.3. Own-Age Effects on Facial Expression Decoding

In this study, we also evaluated the open question of whether own-age effects on decoding accuracy are affected by response bias. For raw hit rates, an own-age advantage in decoding accuracy for disgust and sadness emerged, but it disappeared when response bias was controlled. Moreover, the own-age effects on the proportions of emotion attributions were very weak and only marginally significant. Thus, even the weak response bias may have distorted decoding accuracy, which could explain previous reports of an own-age advantage obtained with uncorrected measures of decoding accuracy [11], revealing the predicted consequence of an increased Type 1 error [12].

### 4.4. Limitations and Outlook

As a possible limitation, the facial expression stimuli used in the present study were relatively short (20 s) video clips. As information processing speed decreases in older age [53], short presentation times may impair performance in older raters. However, the duration of the video clips was comparable to previous studies examining older raters (2-21 s [11], 15 s [14]), and longer video clips might result in more mixed emotional expressions. Nevertheless, future research should examine whether age-of-rater effects on decoding accuracy are reduced when presentation time is extended.

Another promising area for future research is the examination of cultural differences. Previous research revealed effects of ethnic and national group membership on decoding accuracy [54]. Future research may examine whether results of the present study extend to non-Western cultures. For example, stereotypes of aging may differ between Eastern and Western cultures ([55,56], but see [57]), possibly leading to different age-related response bias in Eastern cultures.

A further interesting aim for future research is the examination of age-related differences in facial expressivity. Thus, apart from age-related stereotypes, age-related dialects in emotional facial expressions [58] and habitual emotional facial expressions in older faces [59] may influence the attribution of emotions to older actors’ expressions.

## 5. Conclusions

In the present study, we compared results for unbiased and raw hit rates, analyzing whether age effects remained significant when response bias was controlled. In sum, we found both age-of-actor and age-of-rater response biases. Furthermore, age effects on decoding accuracy for sadness were no longer significant when response biases were controlled. Thus, age-of-actor and age-of-rater effects with regard to decoding sadness may not be fully explained by age effects on the ability to decode sadness, but may be due, at least in part, to response bias. Our results highlight that it is important to consider age-related response bias when analyzing age effects on the accuracy of decoding facial expressions, especially when using spontaneous or ambiguous expressions, both of which lead to lower accuracy and higher guessing rates [12].

## Footnotes

1 WST results were missing for one older rater.

2 Degrees of freedom were corrected due to unequal variances.

3 Because some actors did not give consent to the distribution of their video clips, only a subset of the video clips can be made available to other researchers.
